# Personalized Radiotherapy and Treatment Strategies for Locally Advanced Rectal Cancer: Early Outcomes of a Tailor-Made Total Neoadjuvant Therapy Protocol

**DOI:** 10.3390/cancers18132084

**Published:** 2026-06-26

**Authors:** Atsushi Ogura, Yuki Murata, Yusuke Sato, Shinichi Umeda, Masayuki Tsutsuyama, Tomoki Ebata, Mitsuro Kanda

**Affiliations:** Department of Surgery, Nagoya University Hospital, 65 Tsurumai-cho, Showa-ku, Nagoya City 466-8560, Aichi, Japan

**Keywords:** total neoadjuvant therapy, rectal cancer, nonoperative management

## Abstract

Background/Objectives: The uniform application of total neoadjuvant therapy (TNT) for locally advanced rectal cancer (LARC) risks overtreatment and surgical complications. We evaluated a novel tailor-made therapy that personalizes radiotherapy and chemotherapy to balance oncological safety with organ preservation. Methods: We retrospectively analyzed 38 patients with cStage II–III LARC treated between 2023 and 2025. Patients were stratified by sphincter preservation feasibility and high systemic risk (cN2, extramural vascular invasion, lateral lymph node enlargement). Group A (sphincter-preserving, *n* = 20) received induction chemotherapy; long-course chemoradiotherapy (LCCRT) was omitted in favorable responders but added if MRF-positive or to aim for non-operative management (NOM) in exceptional responders. Group B (non-sphincter-preserving, low systemic risk, *n* = 8) received LCCRT plus consolidation chemotherapy. Group C (non-sphincter-preserving, high systemic risk, *n* = 10) received short-course radiotherapy plus consolidation chemotherapy. Results: Over a median observation period of 20 months (range, 6–37), NOM was initiated in 7 patients (18% overall; Group A: 10%, Group B: 50%, Group C: 10%), with one local regrowth observed to date, resulting in 6 of 7 patients (85.7%) successfully maintaining NOM. Preoperative radiotherapy was safely omitted in 32% of the total cohort, and notably in 60% of patients in Group A. Surgery was performed in 28 patients (74%), achieving an R0 resection rate of 100% across all groups. Distant metastasis recurrence during preoperative treatment occurred in 5 patients (13%). Risk-stratified, tailor-made therapy for LARC facilitates the highly customized application or omission of radiotherapy. Conclusions: Risk-stratified, tailor-made therapy facilitates the safe omission or targeted application of radiotherapy in LARC. This personalized approach prevents overtreatment, maintains complete surgical curability, and achieves successful organ preservation in appropriately selected patients.

## Simple Summary

The standard treatment for advanced rectal cancer often involves radiation before surgery, which can cause significant long-term side effects and may be unnecessary for some individuals. This study aimed to evaluate a personalized treatment strategy to determine if we could safely skip radiation for certain patients. By carefully assessing each patient’s specific tumor characteristics and response to initial chemotherapy, we customized their care plan. We found that this tailor-made approach successfully allowed nearly a third of our patients to avoid radiation therapy altogether, without compromising the complete removal of their cancer during surgery. Furthermore, a subset of patients responded so well that they avoided surgery entirely. These findings are important because they demonstrate that customizing rectal cancer treatment can safely prevent overtreatment, preserving healthy organs and improving patients’ quality of life while maintaining an excellent chance of a cure.

## 1. Introduction

The management of locally advanced rectal cancer (LARC) has undergone a significant paradigm shift over the past decade [1,2]. The traditional approach of neoadjuvant chemoradiotherapy followed by total mesorectal excision (TME) is increasingly being replaced or augmented by total neoadjuvant therapy (TNT) [2]. By delivering both radiation and systemic chemotherapy prior to surgical intervention, TNT was primarily designed to improve systemic disease control, mitigate the risk of distant metastasis, and increase the probability of achieving a pathological complete response (pCR) [3,4,5,6]. Consequently, the enhanced tumor downstaging associated with TNT has accelerated the clinical adoption of non-operative management (NOM) or “watch and wait” strategies, offering highly selected patients the opportunity for organ preservation and the avoidance of significant surgical morbidity [7,8,9,10,11,12].

Despite these oncological advancements, the uniform, standardized application of TNT introduces critical clinical dilemmas. A major diverging and controversial hypothesis in contemporary surgical oncology questions whether maximum neoadjuvant intensity is genuinely necessary for all LARC patients [13]. Intensive radiation and systemic chemotherapy regimens carry substantial risks of functional toxicity, including long-term bowel, urinary, and sexual dysfunction, which profoundly impact a patient’s quality of life [13,14,15,16]. Furthermore, for patients who fail to achieve a complete clinical response and ultimately require TME, the severe pelvic tissue fibrosis induced by intensive TNT can potentially elevate surgical difficulty [17,18,19] and increase the incidence of postoperative complications. Recent landmark investigations, such as the PROSPECT trial, have indicated that preoperative radiotherapy can be safely omitted in specific, well-defined patient subsets without compromising recurrence-free survival [20]. This underscores a growing clinical consensus that the conventional “one-size-fits-all” approach to LARC may lead to overtreatment and is increasingly obsolete.

Therefore, the current frontier in LARC management is the realization of highly personalized treatment pathways [13]. There is an urgent need to establish risk-stratified clinical protocols that carefully balance the potential for sphincter preservation against the risk of systemic recurrence. A tailored approach is required to de-escalate therapy—specifically avoiding unnecessary radiation—in clinically favorable cases, while selectively applying intensive, customized regimens, such as long-course chemoradiotherapy (LCCRT) or short-course radiotherapy (SCRT), only to patients who require maximum local control or who are optimal candidates for NOM [21].

To address this critical clinical gap, our institution implemented a risk-stratified, tailor-made therapy protocol for LARC [22]. The primary purpose of this study is to evaluate the feasibility and early clinical outcomes of this highly personalized approach. We aimed to determine if systematically categorizing patients based on their sphincter preservation potential and the presence of high systemic risk factors (e.g., cN2, extramural vascular invasion, and lateral lymph node enlargement) could safely guide the strategic omission or targeted application of radiotherapy. By shifting away from a uniform TNT pathway, we hypothesize that this customized framework can successfully prevent overtreatment and optimize organ preservation without compromising short-term oncological safety. Ultimately, this study highlights the principal conclusion that the individualized de-escalation of radiotherapy is both a feasible and necessary evolution in modern rectal cancer care.

## 2. Materials and Methods

### 2.1. Study Design and Patient Population

This was a single-center, retrospective observational study conducted at the Division of Surgical Oncology, Department of Surgery, Nagoya University Hospital. The study evaluated a newly implemented, risk-stratified, “tailor-made” total neoadjuvant therapy (TNT) protocol for locally advanced rectal cancer (LARC). The study cohort comprised 38 consecutive patients who were diagnosed with clinical Stage II–III LARC (cT3/4 or N+, within 10 cm from the anal verge) and initiated treatment between January 2023 and December 2025. The study was conducted in accordance with the Declaration of Helsinki, and the protocol was approved by the Institutional Review Board of Nagoya University Hospital (Protocol code: 2021–0136 and date of approval: 28 March 2022). Written informed consent was obtained from all patients prior to treatment initiation.

### 2.2. Risk Stratification and Tailor-Made Treatment Protocol

**Figure 1:**
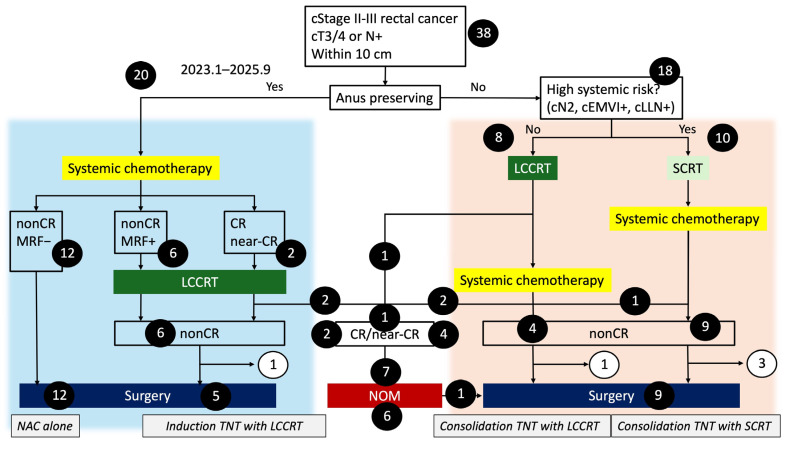
Patient flow.

To balance the goals of optimal organ preservation, oncological curability, and the avoidance of overtreatment, all patients were evaluated by a multidisciplinary tumor board. Treatment pathways were highly personalized based on two primary criteria: (1) the anatomical feasibility of sphincter (anus) preservation, and (2) the presence of high systemic risk factors, defined as clinical N2 disease (cN2), clinical extramural vascular invasion (cEMVI+) [23], or clinical lateral lymph node enlargement (cLLN+). Based on these parameters, patients were prospectively assigned to one of three treatment groups (Figure 1): Group A (Sphincter Preservation Feasible; *n* = 20): Patients in this group received induction systemic chemotherapy as their initial treatment. Following chemotherapy, a primary clinical evaluation was performed. Omission of Radiotherapy: If a patient demonstrated a non-complete response (non-CR) but maintained a negative mesorectal fascia (MRF-), LCCRT (50.4 Gy in 28 fractions with concurrent capecitabine 825 mg/m^2^ twice daily) was omitted, and the patient proceeded directly to surgery. Addition of Radiotherapy: If the patient was MRF-positive (MRF+) [24], LCCRT was administered prior to surgery to ensure local control. If the patient achieved a clinical complete response (cCR) or near-cCR following induction chemotherapy, LCCRT was added with the explicit intent of achieving non-operative management (NOM).

Group B (sphincter preservation not feasible, low systemic risk; *n* = 8): Patients who required a permanent stoma but lacked high systemic risk factors received standard LCCRT followed by consolidation systemic chemotherapy.

Group C (sphincter preservation not feasible, high systemic risk; *n* = 10): Patients who required a permanent stoma and possessed high systemic risk factors (cN2, cEMVI+, or cLLN+) received short-course radiotherapy (SCRT; 25 Gy in 5 fractions) followed immediately by consolidation systemic chemotherapy.

### 2.3. Regimen of Systemic Chemotherapy

Regarding induction chemotherapy in Group A: patients received 6 courses of systemic chemotherapy with either a triplet regimen (mFOLFOXIRI: oxaliplatin 85 mg/m^2^, irinotecan 150 mg/m^2^, leucovorin 400 mg/m^2^, and 5-fluorouracil 400 mg/m^2^ bolus plus 2400 mg/m^2^ over 46 h, every 2 weeks) or a doublet regimen (mFOLFOX6: oxaliplatin 85 mg/m^2^, leucovorin 400 mg/m^2^, and 5-fluorouracil 400 mg/m^2^ bolus plus 2400 mg/m^2^ over 46 h, every 2 weeks) based on patient performance status and clinician discretion. Regarding consolidation chemotherapy in Group B and C: patients received 4 courses of CAPOX (oxaliplatin 130 mg/m^2^ on day 1 plus capecitabine 1000 mg/m^2^ twice daily on days 1–14, every 3 weeks for 4 cycles). Treatment modifications, including dose reductions and schedule adjustments, were performed at the treating physician’s discretion.

### 2.4. Clinical Assessment and Surgery

Clinical response to neoadjuvant therapy was rigorously evaluated using a combination of digital rectal examination, pelvic magnetic resonance imaging (MRI), computed tomography (CT) of the chest and abdomen, and lower gastrointestinal endoscopy. The standardized evaluation of tumor response to induction chemotherapy, which subsequently guided the clinical decision to omit or apply radiotherapy, was based on the criteria previously established in our response-guided strategy protocol [22]; complete response (CR), near-complete response (near-CR), and non-complete response (non-CR). The specific criteria for each category are defined as follows.

CR was defined as satisfying all of the following criteria: (1) endoscopically, a closed ulcer, a linear and flat white scar, and normal wall extension; (2) on pelvic MRI, normalization of the rectal wall or absence of residual intermediate tumor signal with fibrotic low signal on T2-weighted imaging (T2WI), downsizing of involved lymph nodes to a short-axis diameter of less than 5 mm without malignant features (irregular border or internal heterogeneity), and absence of high signal and low apparent diffusion coefficient (ADC) signal on diffusion-weighted imaging (DWI; b1000); (3) smooth and normal findings on digital rectal examination (DRE); and (4) no distant metastasis on computed tomography (CT).

Near-CR was defined as satisfying all of the following criteria: (1) endoscopically, a closed ulcer, an irregular surface with reddish appearance, and decreased—but not normal—wall extension; (2) on pelvic MRI, normalization of the rectal wall or absence of residual intermediate tumor signal with fibrotic low signal on T2WI, downsizing of involved lymph nodes to a short-axis diameter of less than 5 mm without malignant features, and absence of high signal and low ADC signal on DWI; (3) smooth induration or minor mucosal abnormalities without palpable tumor nodules on DRE; and (4) no distant metastasis on CT.

Non-CR was defined as the presence of any of the following: (1) an open ulcer, residual erosion or white moss on the scar, or poor wall extension with submucosal deformity on endoscopy; (2) residual high or intermediate tumor signal on T2WI, lymph nodes with a short-axis diameter of ≥5 mm or malignant features on MRI, or high signal with low ADC signal on DWI; (3) palpable tumor nodules on DRE; or (4) any distant metastasis on CT. For patients who proceeded to surgical intervention, total mesorectal excision (TME) principles were strictly followed.

In this cohort, clinical lateral lymph node enlargement (cLLN+) was defined as a short-axis diameter of ≥7 mm on the primary pre-treatment pelvic MRI, in accordance with criteria established by an international multicenter study [25]. For patients exhibiting cLLN+, ipsilateral LLND was routinely performed following TME, regardless of whether preoperative radiotherapy was administered. Furthermore, for patients with cStage II/III low rectal tumors located below the peritoneal reflection without overt lateral nodal enlargement (short-axis < 7 mm), prophylactic LLND was selectively performed if mesorectal lymph node metastasis was present [26]. However, this prophylactic LLND was intentionally omitted if the patient had received preoperative radiotherapy [27].

### 2.5. Endpoints and Data Collection

The primary endpoints of this early-experience study were the successful omission rate of preoperative radiotherapy, the achievement rate of NOM, and the R0 resection rate for patients undergoing surgery. Secondary endpoints included the incidence of distant metastasis recurrence during the preoperative treatment phase. Patient demographics, tumor characteristics, treatment modalities, and short-term oncological outcomes were extracted from institutional electronic medical records.

### 2.6. Statistical Analysis

Descriptive statistics were used to summarize patient demographics, tumor characteristics, and clinical outcomes. Continuous variables are presented as medians with ranges or interquartile ranges (IQR), depending on data distribution. Categorical variables are presented as frequencies and percentages. Differences between the treatment groups (Group A, B, and C) were evaluated using the Kruskal–Wallis test or Mann–Whitney U test for continuous variables, and the Chi-square test or Fisher’s exact test for categorical variables, as appropriate. A two-sided *p*-value of <0.05 was considered statistically significant. All statistical analyses were performed using [Insert Statistical Software, e.g., SPSS Statistics version 28.0 (IBM Corp., Armonk, NY, USA)].

## 3. Results

### 3.1. Patient Characteristics and Cohort Distribution

Between January 2023 and December 2025, a total of 38 consecutive patients with cStage II/III locally advanced rectal cancer (LARC) were enrolled in the tailor-made therapy protocol. Based on the initial multidisciplinary assessment regarding sphincter preservation and systemic risk factors, the cohort was stratified into three distinct treatment pathways: Group A (sphincter-preserving, *n* = 20), Group B (non-sphincter-preserving with low systemic risk, *n* = 8), and Group C (non-sphincter-preserving with high systemic risk, *n* = 10) (Figure 1). The median observation period for the entire cohort was 20 months (range, 6–37 months). Baseline patient demographics and tumor characteristics are summarized in Table 1.

### 3.2. De-Escalation of Neoadjuvant Therapy: Omission of Radiotherapy

A primary objective of the tailor-made protocol was the safe omission of preoperative radiotherapy to prevent overtreatment. Across the entire cohort, preoperative radiotherapy was safely and completely omitted in 32% (12/38) of patients. This de-escalation was exclusively achieved in Group A; following induction systemic chemotherapy, 60% (12/20) of patients in this sphincter-preserving group demonstrated a favorable response (non-CR, but MRF-negative). Consequently, these 12 patients safely bypassed long-course chemoradiotherapy (LCCRT) and proceeded directly to surgery (Figure 1).

### 3.3. Achievement of Non-Operative Management (NOM)

A clinical complete response (cCR) or near-cCR sufficient to transition to a non-operative management (NOM) strategy was identified in 18% (7/38) of the total cohort, and these patients were enrolled in a watch-and-wait strategy. The initiation of NOM varied across the risk-stratified groups: 10% (2/20) in Group A, 50% (4/8) in Group B, and 10% (1/10) in Group C. At the time of this early analysis (median follow-up, 20 months), local regrowth was observed in one patient from Group B, who received only LCCRT as consolidation chemotherapy was omitted due to severe hand-foot syndrome. Consequently, 6 of the 7 patients who initiated NOM (85.7%) successfully maintained organ preservation without regrowth, suggesting that the rigorous selection criteria effectively identified suitable candidates for this strategy (Table 2).

### 3.4. Surgical Outcomes and Curability

Surgical resection, adhering to TME principles, was ultimately performed in 74% (27/38) of the cohort. The distribution of surgical patients was as follows: 17 patients from Group A, 4 patients from Group B, and 6 patients from Group C. Despite the intentional omission of radiotherapy in a significant portion of Group A, the R0 resection rate (microscopically margin-negative resection) was 100% across all groups and all operated patients (Table 2).

### 3.5. Early Oncological Outcomes and Adverse Events

During the neoadjuvant treatment phase, disease progression manifesting as distant metastasis recurrence occurred in 5 out of the 38 patients (13%). There were no treatment-related mortalities observed during the preoperative induction or consolidation phases (Table 2).

## 4. Discussion

The rapid introduction of total neoadjuvant therapy (TNT) has fundamentally altered the management of locally advanced rectal cancer (LARC), significantly improving systemic disease control and increasing the rates of non-operative management (NOM) and TME-free survival. However, the “one-fit for all” application of intensified preoperative regimens has raised critical concerns regarding overtreatment, functional toxicity, and surgical morbidity. The principal finding of this study is that a tailor-made, risk-stratified clinical pathway is both feasible and oncologically safe in the short-term. By meticulously categorizing patients based on anatomical and systemic factors, we successfully omitted preoperative radiotherapy in 32% of our overall cohort while maintaining a 100% R0 resection rate and initiating NOM in 18% of patients (7/38), of whom 85.7% (6/7) maintained successful organ preservation.

The foundation of our tailor-made protocol is based on the critical distinction that heavily weighs the anatomical feasibility of sphincter preservation against the presence of high-risk systemic features, such as cN2 disease, extramural vascular invasion, and lateral lymph node enlargement. By establishing these distinct parameters upfront, we were able to confidently direct patients with high systemic risk toward intensive regimens while safely de-escalating therapy for those with favorable anatomical and systemic profiles. This stratification prioritizes not only the biology of the tumor but also the patients’ preference in rectal cancer.

Central to this personalized approach is the careful consideration of the pros and cons of radiotherapy. While radiation is beneficial for local disease control and is a cornerstone for achieving a clinical complete response (cCR) aimed at NOM, its long-term functional consequences—including fecal incontinence, sexual dysfunction, and urinary morbidity—are substantial. Furthermore, radiation-induced pelvic fibrosis dramatically increases the difficulty of subsequent surgical resection, resulting in poor local control [17,18]. Our protocol successfully demonstrates that for a specific subset of favorable responders, the functional and surgical drawbacks of radiotherapy can be entirely avoided without compromising the ability to achieve an R0 resection.

Crucially, the safe omission of preoperative radiotherapy, as achieved in a significant portion of our cohort, is fundamentally dependent on two pillars: rigorous patient selection and meticulous surgical execution. Removing the sterilizing effect of radiation inherently shifts the burden of local disease control entirely onto the physical quality of the surgical resection. Without this radiation “safety net” for microscopic residual disease, a precise, high-quality TME becomes paramount to mitigate the risk of local failure. Achieving this rigorous standard within the challenging anatomical confines of the deep pelvis is significantly facilitated by the integration of advanced surgical modalities. Robotic platforms—offering superior three-dimensional visualization and precise instrument articulation—alongside trans-anal or perineal approaches, provide critical technical advantages for securing clear circumferential and distal margins in non-irradiated tissues [28,29]. Furthermore, the technical demands of these advanced modalities, combined with the profound surgical complexity of performing salvage resections for local regrowth following NOM, dictate a necessary evolution in healthcare delivery. The safe and optimal execution of this tailor-made strategy necessitates the centralization of complex LARC management within referral centers or university hospitals equipped with dedicated, expert multidisciplinary teams.

While our protocol has an optional strategy for organ preservation, it highlights the necessity of highly selective criteria for NOM. Although 18% of our overall cohort (7/38) initiated a “watch and wait” strategy, 85.7% of these patients (6/7) successfully maintained organ preservation without regrowth at the time of this analysis, underscoring the importance of rigorous patient selection. Nevertheless, this approach must be pursued with extreme caution. Recent literature indicates that patients who experience local regrowth following TNT subsequently require salvage surgery which is technically demanding [17,18,19] and often suffer a poor prognosis [30]. Therefore, in our cohort, NOM was not offered as a blanket option but was rigorously restricted to patients achieving a strict cCR or near-cCR. Balancing the profound quality-of-life benefits of organ preservation against the oncological hazards of local regrowth remains one of the most delicate decisions in modern rectal cancer care.

The distant metastasis rate of 13% observed during neoadjuvant treatment in our cohort warrants contextualization against landmark TNT trials. In the RAPIDO trial, the 3-year rate of disease-related treatment failure (which includes distant metastasis) was 23.7% in the SCRT plus consolidation chemotherapy arm versus 30.4% in the standard LCCRT arm, reflecting a long-term cumulative incidence rather than an event rate during the preoperative phase [3]. In PRODIGE-23, the 3-year distant metastasis-free survival was 75.7% in the FOLFIRINOX plus CRT arm versus 68.5% in the standard CRT arm [4]. In the PROSPECT trial—which, like our protocol, evaluated selective omission of preoperative radiation in a subgroup—the 5-year distant metastasis rate was approximately 19% in the chemotherapy-alone arm [20]. The 13% distant metastasis rate detected during preoperative treatment in our cohort is therefore relatively high for this phase of therapy, though it should be noted that our patients include a high proportion with aggressive systemic risk features (cN2, cEMVI+, cLLN+): 74% had cN+ disease, 42% had cEMVI+, and 21% had enlarged lateral lymph nodes. This underlying high-risk biology, together with the short follow-up period, may account for the higher early distant failure rate and merits close monitoring in future follow-up.

Despite these promising early outcomes, this study has several limitations. First, the sample size is small (*n* = 38), and the study design is a retrospective, single-center analysis, which may introduce selection bias. Second, the overall median observation period of 20 months is insufficient to evaluate long-term oncological outcomes, such as 3-year TME-free survival, overall survival, or late pelvic recurrence. Third, this study lacks a contemporaneous control group receiving standard TNT or conventional LCCRT, which precludes direct comparison of outcomes and prevents definitive conclusions about the superiority or non-inferiority of the tailor-made approach. The absence of a control arm means that observed R0 resection rates and NOM rates cannot be attributed solely to the protocol itself. Fourth, this early-experience report lacks patient-reported outcome (PRO) data, including bowel function assessments (LARS scores [31]), urinary function, sexual dysfunction metrics, and quality-of-life questionnaires. Given that a primary rationale for selective radiation omission is to reduce treatment-related functional morbidity, the absence of such data represents a significant gap. Similarly, comprehensive reporting of neoadjuvant treatment-related toxicities (grade 3–4 adverse events) and treatment compliance rates is limited in this early report and should be prospectively collected in future studies. Future prospective trials with larger cohorts and longer follow-up periods are essential to validate these initial findings and comprehensively assess the functional benefits of selective radiation omission.

## 5. Conclusions

In conclusion, our early experience demonstrates that a risk-stratified, tailor-made therapy protocol for locally advanced rectal cancer is both feasible and oncologically safe in the short-term. By systematically stratifying patients according to sphincter preservation feasibility and systemic risk burden, preoperative radiotherapy was safely omitted in 32% of the cohort while maintaining a 100% R0 resection rate and initiating NOM in 18% (7/38), with 85.7% of those patients (6/7) successfully maintaining organ preservation. Notably, this study does not include a control group or functional outcome data; prospective studies incorporating patient-reported outcomes and longer follow-up are needed to fully validate this approach. These findings highlight that individualized application or omission of radiotherapy represents a necessary evolution in modern rectal cancer care, fundamentally relying on rigorous patient selection and meticulous surgical execution within specialized multidisciplinary centers.

## Figures and Tables

**Table 1:** Patient and tumor characteristics.

Total Cohort	Total (*N* = 38)	Group A (*N* = 20)	Group B (*N* = 8)	Group C (*N* = 10)
Age (IQR)	63 (51–72)	60 (49–70)	66 (44–72)	62 (60–72)
Sex (man)	23 (61)	13 (65)	5 (63)	6 (60)
Distance from anal verge (mm, IQR)	40 (30–66)	64 (40–78)	36 (29–44)	30 (21–39)
cT stage 2/3/4	2/19/17	0/11/9	2/5/1	3/7
cMRF involvement	24 (63)	11 (55)	4 (50)	9 (90)
cN+	28 (74)	18 (90)	2 (25)	8 (80)
Extramural vascular invasion	16 (42)	9 (45)	0	7 (70)
Swollen lateral lymph node (SA ≥ 7 mm)	8 (21)	5 (25)	4 (50)	4 (40)
Regimen of chemotherapy, Triplet	22 (58)	17 (85)	1 (13)	4 (40)
Duration of chemotherapy (weeks, IQR)	12 (12–12)	12 (12–15)	12 (10–12)	12 (12–18)
Regimen of radiotherapy, LCCRT/SCRT	6/1	2/0	4/0	0/1
Omission of radiotherapy	12 (31)	12 (60)	0	0
Differentiation, well/mod	33 (87)	19 (95)	8 (100)	6 (60)
RAS status wild/mutant	15/23	9/11	3/5	3/7
BRAF status wild/mutant	37/1	20/0	7/1	10/0
MSS/MSI	38/0	20/0	8/0	10/0
Distant metastasis during TNT	5 (13)	1 (5)	1 (13)	3 (30)

IQR: interquartile range, MRF: mesorectal fascia; SA: short-axis; LCCRT: long-course chemoradiotherapy; SCRT: short-course radiotherapy; TNT: total neoadjuvant therapy.

**Table 2:** Outcomes of nonoperative management and surgery.

Total Cohort	Total (*N* = 38)	Group A *(N* = 20)	Group B (*N* = 8)	Group C (*N* = 10)
Nonoperative management	7 (18)	2 (10)	4 (50)	1 (10)
Local regrowth	1 (14)	0	1 (25)	0
Surgery cohort	*N* = 27 *	*N* = 17	*N* = 4	*N* = 6
Operative procedure (LAR/APR/TPE)	10/10/7	9/6/2	1/3/0	0/1/5
Lateral lymph node dissection	14 (52)	9 (53)	0	5 (83)
Total operative time (min, IQR)	544 (352–710)	544 (365–645)	324 (289–370)	711 (541–729)
Total blood loss (mL, IQR)	85 (36–235)	77 (36–221)	62 (16–223)	167 (90–393)
pT stage (Complete response/2/3/4)	2/7/15/3	2/3/11/1	0/3/1/0	0/1/3/2
pN stage (+)	9 (33)	5 (29)	1 (25)	3 (50)
Lateral lymph node metastasis	4 (15)	3 (17)	0	1 (17)
R0 resection	27 (100)	17 (100)	4 (100)	6 (100)
Tumor regression grade (Dworak’s) 1/2/3/4	7/12/6/2	4/6/4/2	2/2/0/0	1/3/2/0
Severe postoperative complications (CD ≥ 3)	10 (37)	6 (35)	1 (20)	4 (40)
Anastomotic leakage	1 (4)	1 (6)	0	0
Postoperative hospital stay (days, IQR)	23 (16–29)	23 (15–24)	17 (15–20)	32 (27–45)
Local recurrence	0	0	0	0
Distant recurrence	2 (7)	2 (12)	0	0

NOM: nonoperative management; LAR: low anterior resection; ISR: inter-sphincteric resection; APR: abdominoperineal resection; TPE: total pelvic exenteration; CD: Clavien–Dindo; * including one salvage surgery for local regrowth.

## Data Availability

Further data could be submitted on request according to the institutional rules of patient’s data management.

## Abbreviations

The following abbreviations are used in this manuscript:

LARC	Locally advanced rectal cancer
TME	Total mesorectal excision
TNT	Total neoadjuvant therapy
cCR	Clinical complete response
pCR	Pathological complete response
NOM	Nonoperative management
LCCRT	Long-course chemoradiotherapy
SCRT	Short-course radiotherapy
EMVI	Extramural vascular invasion
LLN	Lateral lymph node
MRF	Mesorectal fascia
MRI	Magnetic resonance imaging
CT	Computed tomography
IQR	Interquartile range
SA	Short-axis
MSS	Microsatellite stable
MSI	Microsatellite instability
LAR	Low anterior resection
APR	Abdominoperineal resection
TPE	Total pelvic exenteration
CD	Clavien–Dindo (classification)
LARS	Low Anterior Resection Syndrome
